# A rare case of trauma related dissociative identity disorder

**DOI:** 10.1192/j.eurpsy.2023.2030

**Published:** 2023-07-19

**Authors:** F. Ghrissi, F. Fekih-Romdhane, M. Stambouli, B. Abassi, M. Cheour

**Affiliations:** 1 Razi Hospital, Mannouba, Tunisia; 2 psychiatry department E, Razi Hospital, Mannouba, Tunisia

## Abstract

**Introduction:**

Dissociative identity disorder (DID) is a debilitating and controversial psychiatric disorder with a lifetime prevalence estimated around 1,5%. It remains underdiagnosed despite recognition in international classification of mental disorders. In fact, based on the DSM-5 criteria, DID is characterised by two or more distinct personality states that coincide, with fluctuating consciousness and changing access to autobiographical memory. The aetiology of DID has long been debated with recent neuroimaging evidence supporting the trauma model of this condition.

**Objectives:**

The aim of this presentation is to describe the case of a young female diagnosed with DID related to childhood trauma.

**Methods:**

We also conducted a literature review in order to discuss the aetiology of the disorder. The following keywords were searched through the pubmed website: dissociative identity disorder, trauma, aetiology.

**Results:**

We report the case of a 20 years old female with no past medical, nor psychiatric history. However, she had a family history of an uncle and an aunt with chronic psychosis. Her father died when she was 8, thus she lived with her mother and her brother and two sisters. She was a brilliant student and started engineering studies. She has no particular personality trait. She was raised within a strict religious family with little time dedicated to leisure activities. Importantly, since the age of 10, she was exposed to her mother’s religious extremist and threatening discourses, related to death and “grave’s torture” and comprising many cultural beliefs. She seeks for psychiatric care complaining of “soliloquy” that became remarkable by her relatives. On psychiatric evaluation she presented daily fluctuating consciousness during at least one hour, in which she switches identity toward the daughter of a famous singer. This alter was having pleasant activity with her mother and was singing and hanging out most of the time. No particular triggers were identified. The trouble started by the age of 14 then worsened gradually and became an unvoluntary phenomenon with significant distress. She had no depressive nor psychotic nor anxiety or obsessive symptoms. Her sleep and appetite were not disturbed. She met DSM-5 diagnostic criteria for DID and was referred to a trained psychiatrist for adequate psychotherapy management.

**Conclusions:**

We exposed a rare case of a young student complaining of soliloquy since the age of 14 that was diagnosed with DID subsequent to a particular childhood trauma which consisted in exposure to threatening religious and cultural beliefs about life after death told by her mother. This unique case emphasises the trauma model of DID, where the nature of the trauma influences the clinical expression of DID. Given the recent neuroimaging evidence, DID can be framed as a chronic psychiatric disorder based on neurobiological, cognitive, and interpersonal non-integration as a response to unbearable stress.

**Disclosure of Interest:**

None Declared

